# The body–space relations of research(ed) on bodies: The experiences of becoming participant researchers

**DOI:** 10.1111/area.12367

**Published:** 2017-07-10

**Authors:** Emma Wainwright, Elodie Marandet, Sadaf Rizvi

**Affiliations:** ^1^ Institute of Environment, Health and Societies Brunel University London Uxbridge Middlesex UK; ^2^ Faculty of Wellbeing Education and Language Studies Walton Hall Kents Hill Milton Keynes UK

**Keywords:** body work, embodiment, field notes, methodology, participant research, training

## Abstract

This paper heeds calls for reflections on how the research field is defined through embodied socio‐spatial presence and immediacy. Focusing on classroom “body‐training” observations that were part of a larger qualitative research project, and on the field notes and reflections of three researchers, we explore the transition from observer‐researchers to participant‐researchers. That is, we explore how, by researching others, we unexpectedly became researched on as our own bodies became instruments in the research process and were used to elicit knowledge on embodied learning, body‐mapping and corporeal trace. As a methodological intervention, conducting research through the body, the positioning of bodies and body‐to‐body interaction, can tell us much about the often ignored embodied and emotional dimensions of the research field. But, in addition, it can elucidate the power relations between, and the fluidity of, researcher and researched positions in the jolting of secured researcher identity. Here we detail how different researchers performed different embodied and emotional subjectivities in different training research spaces. We explore how ontological anxieties of our own placed bodies, based around constructed notions of femininity, religion and researcher professionalism, shape this immediate body‐to‐body encounter and the subsequent research process.

## 1 INTRODUCTION

In recent years, what Crang referred to as the “ghostly absence of researchers” bodies in the research process’ (2003, p. 494) has been confidently attended to in geographical inquiry, notably in feminist and queer geographies (Bain & Nash, 2006; Longhurst, 2001; Longhurst & Johnston, 2014). Longhurst et al.'s (2008) recognition of the body as an “instrument of research” is critical (see also Al‐hindi & Kawabata, 2002), requiring our positioning in relation to, for example, race, age, gender, but also highlighting other important bodily aspects such as smell, reactions, clothing and touch that need to be brought to the fore. It is these other embodied dimensions of working so closely with others’ bodies that are too often missing from current research and to which this paper attends in a particularly immediate way through unexpected and one‐off participant observations. We argue that it is vital to acknowledge our bodies, these other bodily aspects and the spatial relations between bodies – what we refer to here as body–space relations – to understand the dynamics of the research process more fully. This serves as a reminder that researcher identity is never secure, bounded and controlled.

Drawing on field notes^1^ and reflections of three differently positioned women academics engaged in class observations, we focus here on the transition from observer‐researchers to participant‐researchers. These observations were one part of a larger research project on training for body work which also involved interviews, focus groups and participant diaries. By researching others’ bodies through observations, we focus on how we unexpectedly became “researched on” as our own bodies became instruments in, and vectors of, the research process and were used to elicit knowledge on embodied learning. As a methodological intervention, conducting research through the body, the spacing of bodies and body‐to‐body interaction, can tell us much about the often ignored embodied and emotional dimensions of the research field. In addition, we elucidate the power relations between, and the fluidity of, researcher and researched positions in the jolting of secured researcher identity. As researchers, we do not readily admit discomfort and concern, especially when it is deeply personal and related to our own embodied presence. Here we detail how different researchers performed different embodied and emotional subjectivities in different training‐research spaces. We explore how ontological anxieties of our own placed bodies, based around constructed notions of femininity, religion and researcher professionalism, shape this immediate encounter and the research process.

Taking the lead from the paper's title, our discussion is structured as followed. First, and by way of context, we discuss *research on* bodies and our wider “body‐training” project. That is, our research on others’ bodies, linked to concepts of body work and embodied labour, and their resonance for understanding the training/learning process and environment. Second, and more substantively, we explore how we became *researched on* bodies through one‐off unintended encounters as “guinea pigs” in the training process as, fleetingly, we became corporeally present participant researchers. The usefulness of research through the body, the body as a site of knowledge production, and the immediate body‐to‐body encounter are considered. Alongside this, we question researcher identity; that is, our own variously situated embodied and emotional subjectivities and responses to being researched on bodies, and our ontological anxieties around this. We are mindful of what Wacquant says about the need for a “sociology not only of the body, in the sense of object, but also from the body, that is deploying the body as a tool of inquiry and a vector of knowledge” (2004, p. viii). We consider how thinking about the body as vector through which meanings and understandings are transmitted and experienced (Buckingham & Degen, 2012) creates knowledge about ourselves, our identities as researchers and perceived impacts on research relationships. In short, it can tell us as much about ourselves as our research participants, and reflecting on this is necessary for a fuller appreciation of the research process. Third, and by way of conclusion, we reflect on the body–space relations between researchers and researched through such immediate embodied encounters, referring to core methodological concerns of power relationships, positionality and reflexivity that geographers have long explored. It is not enough for researchers to simply state upfront the categories to which they belong and the positions (of difference and sameness) they hold (Bain & Nash, 2006; Ellingson, 2006). Instead, we argue for fuller reflection on the fluid and more corporeally charged nature of doing research through immediate body‐to‐body encounters, and why this matters.

## 2 RESEARCH ON BODIES

The conceptual territory of our research lies foremost in the literatures relating to the pervasive gendering of body work and emotional labour, and the recursive relationship between the spaces and experiences of learning bodily and emotional skills for transfer to paid employment. Here we sketch out these ideas in relation to our larger research project to foreground embodied learning and position us in the research process.

Body work, as elucidated by Wolkowitz (2006), is defined as work involving the interaction between bodies; work that is often intimate and messy, and based on touch and close proximity. It has been conceptualised through highly sexualised, gendered and maternalised discourses of motherhood, care and familial responsibility (Twigg, 2000) and a pervasive sexual division of labour that assigns women predominantly to the care of bodies and spaces they inhabit (Oerton, 2004). Through essentialist and performed (self)constructions of women's abilities, it is “intimately linked with women's bodily lives through motherhood and nurturance” (Twigg, 2000, p. 407). Furthermore, drawing on Hochschild's formative writings (1983), body work has been explicitly related to emotional labour that it frequently requires (Gimlin, 2007). For example, Twigg (2000) and Milligan (2003) have drawn attention to both the physicality and emotionality of the caring dimension of body work, pointing to how care is implicitly entangled in gendered meanings of home and identity.

The project from which this paper comes was a 2.5‐year study exploring what we called the “body training” choices, expectations and experiences of mothers in the West London area.^2^ It focused on training courses popular with mothers – that is, training in areas of, and for, body work. The research had a strong policy dimension with the persistent encouragement of women, notably mothers, into training for (re)employment. In addition, one key research question focused on how women learn and negotiate the embodied physicality and emotionality of body training. To do this, the project examined training in and for a number of different areas of body work loosely conceptualised by Wolkowitz (2006) as caring, curing, pleasuring and adorning, including childcare, massage, reflexology, nursing and hairdressing.

In terms of our research on bodies and as a research team, we have been interested in the spatial dynamics of training for body work. We have variously discussed the confluence of “professional” discourses with those pertaining to normative constructions and performativity of gender and maternal identity, the role of embodied discipline and the process of learning, manipulating and using space in the training process (Wainwright & Marandet, 2015; Wainwright et al., 2011; Wainwright et al., 2010). From these substantive papers, our own role as embodied researchers kept provoking us; as we were writing about and reflecting on the bodies of others in the learning process, should we not also reflect on our own bodies and what we learned through and about our bodies in the practice of research? The rest of the paper takes this as its lead in relation to becoming researched on bodies.

## 3 RESEARCHED ON BODIES

### 3.1 Learning through bodies

It has been through sustained scholarship in the fields of feminist and queer geographies that research through the body has been highlighted and interrogated. In a paper on embodiment and sexuality at a queer bathhouse, Bain and Nash (2006) emphasise researchers’ bodies as a vital tool for data collection. Through close ethnographic fieldwork, they reflect on how the researching body was dressed, positioned and socialised and hence destabilised. Led by feminist challenges to masculinist knowledge production and power relations, their research is a significant addition to researching others’ bodies and embodying the research context: “The researching body … cannot be understood as stable or fixed; rather it needs to be rendered explicitly visible as a contested site of knowledge production” (2006, p. 99).

We reiterate this need for visibility but extend the analytical lens to focus on reflections on direct embodied encounters and the ontological pleasures and awkwardness that extend from our overt research encounters. Field notes of our embodied, often visceral, and emotional experiences of being worked on and over, form the basis of this section, and tally with a critical self‐reflective research approach (England, 1994).

Three of our classroom‐training observations presented us with the opportunity to move from detached observer‐researchers to participant‐researchers. These encounters were unplanned and unexpected as we were used as bodies to be “researched on” by participants to make up class numbers. For Sadaf, this was in relation to hairdressing, Elodie, aromatherapy massage, and Emma, full body massage. Though brief, constituting the practical part of one teaching session, these guinea‐pigging opportunities gave insight to the processes of training and embodied learning. Importantly, these were one‐off fleeting and “unanticipated” encounters, and not sustained participation as has been discussed elsewhere, including by Tarr (2011) on learning Alexander Technique, Lea (2009) on learning yoga, Buckingham and Degen (2012) on teaching yoga, and Gale (2009) on researching osteopathy and homeopathy. In common with this growing work, it gave us a different awareness of the embodied and emotional aspects of the learning process and the usefulness of researching *through* the body.

We also need to say something of ourselves at this point. Though all researchers, we were positioned differently in relation to research participants and one another. When research was conducted, we were all in our early/mid‐thirties and held or were completing doctorates, in contrast to the women we were researching who were undertaking National Vocational Qualifications at level 2 or 3 in further education colleges. We are all heterosexual and, at the time of research, only one of us was a mother with the other two subsequently becoming so. Elodie is white French, Emma white British and Sadaf Pakistani. Additionally, only Sadaf could be identified by religion through the wearing of the *hijab*. So, while there were points of commonality between us and the mothers we researched, there were also points of difference, some of which only became apparent through the practice of research.

Our field notes pondered different aspects of this body‐to‐body interaction, both the deeply corporeal trace of body training, but also our embodied affect:

The pressure exerted by the tutor as her hands moved across my back was in stark contrast to the lighter hesitant touch of the student – my body ached with displeasure and I was pleased when she'd stopped. (Emma)

My hair was pulled several times which was painful, a sensation which was felt through my body. (Sadaf)

For Emma, the student's lighter hesitant touch, with her back being cautiously and carefully mapped and massaged as the student negotiated her notes, the fleshy body and tutor gaze to ensure she worked on the correct constituent parts, contrasted with the forcefulness of the tutor's well‐trained hands. This gave prized insight into what the students were trying to achieve and the spatial ordering of one body in relation to another (Lea, 2009) in the training process. Sadaf, with her long “unruly” hair and the challenges it seemed to present, noted the student's lack of technique and care that made for a viscerally painful, wincing encounter. The very materiality of our bodies was brought to the fore with our affective and emotional reactions etched on faces, producing tensed forms. On reflection, we wondered how far our research participants could have known, felt and read our affective unease from and through our bodies.

Such reflections tally with Lea's (2009) assertion that skill emerges when “tightly coupled assemblages” of bodies, techniques, objects, contexts and knowledges are formed. This was especially prescient in the body‐training classroom with our experiences enabling us to understand the processes by which skill is learned and how new bodily capabilities are unevenly and haphazardly developed. As in Wacquant's (2004) research, our bodies became important instruments of research, used to gain insight into the research subjects and training process. But what did this mean for our researcher identity and what were their implications for the research process?

Embodiment and emotions are not just tied to individual bodies but bound up with wider structures and processes, and these were played out through our responses in situ. The body cannot be understood as and of itself but is constructed through and by social and cultural discourses, and worked through the materiality of the body. In our research, we raised ontological anxieties and pleasures of being researched on bodies which were variously linked to constructed performances of femininity, religion, and researcher professionalism. By thinking through how we negotiated our embodiedness, in relation to our bodies as unprepared, un‐groomed and (dis)comforted, we questioned our very selves in the research process.

### 3.2 Unprepared bodies

Having travelled by public transport, we arrived at our evening training observations from a day's work in what we describe as informal professional work attire. Our bodies were tired, and Elodie and Emma especially, as they had to remove clothes for massage, were concerned they would reveal smelly, dirty and sweaty bodies; smell, sweat and dirt that was otherwise hidden and out of sight, maintaining researcher respectability:

At that moment, I was relieved to have a bit of time on my own to check myself: was I not too sweaty or smelly. (Elodie)

I felt exhilarated yet apprehensive. I wasn't prepared for this. I had been in these clothes all day. Would my body be acceptable? Was it too dirty? (Emma)

Temporalities and spatialities of our day's labour and journeying were marked on and discernible through our bodies with concerns over both how we were experiencing, and how others would experience, them. With nakedness at odds with social mores and expectations, our clothed professional academic identities were disrupted. As England highlights, the “researcher cannot conveniently tuck away the personal behind the professional, because fieldwork is personal” (1994, p. 85). Our clothes and demeanour are markers of professional status and suddenly these were lacking. Douglas's (1980) work on dirt and more recent research by MisGav and Johnston (2014) on the materiality of sweating bodies as matter out of place, are pertinent here. Bodies are deemed dangerous when they leak into or onto other spaces and bodies. Sweating bodies go against ideas of femininity, with women spending a great deal of time ridding themselves of sweat and dirt to ensure they are considered attractive and acceptable. Longhurst (2001) has argued that bodily fluids are part of a larger discourse employed to maintain a masculine/feminine binary. These discourses were played out in our reflections of our nakedness and perceived embodied acceptability, and we performed a version of femininity where clean, clothed and prepared bodies are necessary, most especially for the research process and to ensure appropriate researcher identity. Our own bodies were leaking onto and into our clean, neat and organised research process in ways we had not prepared for, disrupting our efforts to maintain “respectable” researcher professionalism.

### 3.3 Un‐groomed bodies

This sense of unpreparedness was reiterated in relation to grooming, and presenting as “un‐groomed” bodies. In the quotes below, there is a degree of loathing of our bodies as being deficient in some way. Not anticipating this type of research engagement, we had not discussed this and readied ourselves in advance, in contrast to others engaging in embodied research (Bain & Nash, 2006). As Waitt (2014) expresses, self‐disgust maintains spatial, gendered and moral dimensions of individual subjectivities. Linked to an expected femininity, and set of perceived feminine behaviours and embodied expectations, we all felt deficient. Indeed, it was this unexpected opportunity that gave us time and immediacy to reflect on our bodies in a way we were perhaps unused to. This is not what we had expected in our researcher role and we recount a visceral shame/embarrassment in our embodied presentation. In particular, we were alert to our embodied difference with the clean, well‐groomed, trained, professional “in‐the‐making” bodies around us:

I'm not ‘groomed’ like the bodies in the massage class. My nails are bitten, legs not recently shaved and I worried that my dermatographia^3^ makes my pale white skin red and unsightly when massaged. (Emma)

As I looked at myself in the mirror, I suddenly realised how tired and ‘unfresh’ I looked; how a double chin was growing down my face and how much scope there was for improvement in my look. (Sadaf)

My legs are covered in thread veins which make them look like they belong to someone twice my age. (Elodie)

A self‐disciplinary gaze worked on us, marking us as “other” to the expected neat, trim, professional and uniformed bodies around us. Our ontological anxieties around failure to perform socially appropriate feminine respectability led to visceral shame and ignominy through which we experienced the embodied learning process, and was carried later with us as we reflected on our experiences.

### 3.4 (Dis)comforted bodies

Geographers have noted the issue of subjective (dis)comfort in the doing of research in numerous ways, most notably in relation to emotional geographies (Pile, 2010; Punch, 2012). We experienced differing embodied and affective responses to our participant observations as we were differently scrutinised. For Elodie, comfort translated into feelings of relaxation and being soothed:

She also massaged my scalp…. I felt incredibly relaxed and blissful and a bit spaced out. (Elodie)

For Emma, discomfort was translated through feelings of awkwardness and not knowing how to “be”:

Massage makes me feel uncomfortable: I felt my body tensing, my heart‐racing, feeling awkward, as well as cooling down without my clothes. (Emma)

As researchers, we aim to put participants at ease, encouraging them through our research techniques and personality (Moser, 2008) to speak and share experiences. We suddenly found ourselves the ones being studied, an inversion of the typical power relationship of research, a position to which we had differing embodied responses.

For Sadaf, discomfort was brought into sharp focus in another specifically religiously constructed and embodied way:

She [the tutor] examined how my hairdresser had done her job and gave her instructions to do a little more trimming so that the hair looked healthier. At this point, a boy walked into the room. He was the only male student in the course and was away in the morning. I suddenly became concerned about my privacy as I was not wearing my *hijab*. It made me feel uncomfortable. Moreover, another man entered the college building and sat outside the salon on a sofa where he could see through the glass walls of the salon. (Sadaf)

As a Muslim woman who wears a *hijab*, Sadaf was left feeling visible and exposed, wary of her environment and disrupting the process of participant observation due to awareness of her embodied presence in relation to men. Her hair, which is part of her *awrah*,^4^ was at risk of being shown to men who were clearly oblivious of her values and explicit discomfort. The dismantling of her identity through moving from observer‐researcher to become an object of training placed her in a dilemma of how to negotiate between the social norms of politeness and researcher professionalism and her own religious observance.

These examples demonstrate differing, perhaps opposing, notions of respectable femininity coming into play for each of the researchers. Lupton's (1998) ideas on emotions as intersubjective rather than individual, and so constituted in the relations between people, come clearly into play, with ontological anxieties around research spaces and bodies. For Sadaf, her emotional response and anxieties were borne out of spatial closeness of those in the training space with difference constructed and experienced through the body. Despite the awareness of potential difficulties that taking on the role of participant researchers may invite, it is hard to anticipate and, more so, deal with the dilemmas that emerge in relation to our own unexpected and fleeting, but spatially immediate, embodied co‐presence in research.

## 4 CONCLUSIONS: BODY‐SPACE RELATIONS

All researchers have bodies that should be acknowledged (Ellingson, 2006) and in this paper we have reflected on how our bodies unexpectedly became foregrounded in the doing of research and how this disturbs the research process. Through conducting research on others’ bodies, we became researched on bodies ourselves through classroom interactions that involved direct touch, embodied repositioning and corporeal immediacy. The opportunity to “guinea‐pig” in practical training sessions was useful but also unnerving, forcing us to question the role and place of our own bodies in research. Punch (2012) writes about why, as researchers, we can be so apprehensive about being open and honest in relation to revealing extracts from field notes. Here, we have done this, but with trepidation and a sense of vulnerability given their personally embodied and corporeal nature. Through an appraisal of our perceived embodied (un)preparedness, (lack of) grooming and (dis)comfort, we have shown that research on body work can have problematic and even painful impacts.

Academics have traditionally viewed their bodies as what carries their minds or intellects. Researcher identity is normatively a cognitive construct, rather than an embodied one (Turner & Norwood, 2013) and though geographers are attending to their own bodies in the practice of research, we argue that this can be taken further. Dyck (2002) has argued that we need to think more about the embodied researcher whose interpretations tell a story of others’ lives and embodiment. We would add to this that it also tells us something important about our own lives, identity and subjectivity as professional researchers, and thus the research we do. Embodiment is often acknowledged but not detailed, full and immediate, and there is more to be written on the practices and experiences of the embodied researcher. For us too, this has been an ethical dilemma in our research; as we observe, and think, talk and write about, others’ bodies, should we not also consider more fully the place, materiality and effect of our own bodies? This raises questions of power, hierarchies and reciprocity, but also benefits. Our “guinea‐pigging” benefited research participants (they had free models to work on), but it benefited our research process and understanding of embodied practice as we could better observe and understand body‐training experiences.

Reflexivity has been given great coverage, especially in relation to emotions and personality (Moser, 2008; Punch, 2012). Using field notes, it is common for researchers to reflect on their experiences of doing research, thinking through subjectivities based on gender, race, class and concomitant positioning of insider/outsider. Punch (2012) discusses how emotions mediate the research process, but so too can the materiality of our bodies, which is too often left out or relegated to the periphery. For us, reflections on our “imperfect” bodies and embodied uneasiness heightened a sense of apprehension in allowing our bodies to figure in our research and writing. Yet, ontological anxieties and pleasures about becoming researched on bodies, and mediated through our personalities, has forced us to shake out the mind–body dualism so forcefully present in research.

As a legacy of feminist writing has demonstrated, researchers are not all knowing (Laurie et al., 1999) and as such we cannot know what impact our very embodied presence and experience had on our participants and the research process, and this could be attended to in future research. Yet our bodies are active participants in the research process, and being worked on, and being seen to be worked on, loosened later research encounters in focus groups and in‐depth interviews. This closeness and familiarity with research participants in an immediate and embodied sense has a bearing on power dynamics. Having our research participants either run their hands through our hair or over our semi‐naked bodies, or see this being done to us, did make us reflect on the dynamism of power relations in the research process as we were no longer detached observer‐researchers. For all of us, this led to a sense of an easing of research relations, as Elodie reflected: “it made it impossible for her to see me as remote and intellectual”. This embodied encounter opened us up as researchers, exposing us and enabling a different kind of “knowing”. We could later use our researcher experiences to discuss, even laugh about, our embodied participation in their training courses and initiate research conversations. The usual distance and hierarchy between the researcher and the researched, which often persists despite our best efforts, was changed and narrowed in the later interviews, enabling easier research connections to form (Sharma et al., 2009) and discussions on the intimacies/uncertainties of touch in body‐training.

As a final point, we want to stress that body–space relations between researcher and researched do matter, and that research practice and the process of research is about more than what is said. We need to attend to the sometimes personal and intimate, as a focus on bodies of the researched and researchers’ bodies and the spaces in between, destabilises the rational and abstract, detached and too often linear, accounts of the research process. This points to complexity and vulnerability in the doing of research and researcher position, moving to a co‐production of research and generation of knowledge, through a shifting of power relations. In such a way, the doing of research can be considered a form of body work requiring critical (self)reflection and dialogue.

## ENDNOTES

1 Though we use the term field notes, we are mindful of the distinction made between field notes and field dairies. As Punch (2012) differentiates, field notes describe what is happening in the field, whereas field diaries record how the researcher feels about the research process. Our field notes included both elements as described by Punch.

2 The research was funded by the ESRC (First grants scheme no. RES‐061‐23‐0106). Further details can be found at http://www.esrc.ac.uk/my-esrc/grants/RES-061-23-0106/read.

3 Dermatographia is a condition whereby when skin is scratched or rubbed it reddens and becomes raised.

4 For Muslim women, *awrah* (the parts of body which should not be revealed to unrelated men) is her entire body except face, hands and feet.
